# Supplementary material for: Marrying for power: Gendered alliances in mafias
Source: PLoS One. 2026 May 6;21(5):e0345859. doi: 10.1371/journal.pone.0345859 (PMC13148698; doi:10.1371/journal.pone.0345859)
Supplement: Attachment — Submitted filename: answers_to_the_reviewers.pdf [file pone.0345859.s002.pdf]

PONE-D-25-40694

Marrying for Power: Gendered Alliances in Mafias

PLOS ONE

Thank you for submitting your manuscript to PLOS ONE. After careful consideration, we feel that it has merit but does not fully meet PLOS ONE's publication criteria as it currently stands. Therefore, we invite you to submit a revised version of the manuscript that addresses the points raised during the review process.

The manuscript examines matrimonial alliances among 'Ndrangheta families using judicial sources and a clan-level marriage network, highlighting the centrality of powerful clans, the role of peripheral-to-peripheral marriages, and the balance in bride reception. While the topic and dataset are valuable, several methodological issues require clarification: modeling clans as nodes oversimplifies the network and ignores individual-level dynamics; excluding intra-clan marriages removes information on endogamy and internal cohesion; ignoring network directionality limits analysis of gendered exchanges; some figures are difficult to interpret and could be moved or replaced; robustness procedures and the classification of "elite" versus "peripheral" clans need more transparency; potential biases in judicial data should be discussed in greater detail; and the manuscript could be streamlined, references refined, terminology standardized, and data availability described more clearly to improve clarity and reproducibility.

Dear Prof. Porcher,

We wish to express our sincere gratitude to you and the reviewers for the thoughtful and constructive feedback provided on our manuscript. We are particularly encouraged by the positive assessment regarding the value of our dataset and the importance of the study's contribution to the understanding of criminal networks.

In accordance with the editorial recommendations and the specific points raised during the review process, we have implemented a comprehensive revision of the manuscript. A primary focus was the refinement of our modeling framework; we have now provided a robust sociological and empirical justification for adopting the clan-level unit of analysis, while simultaneously incorporating intra-family endogamy as a distinct strategic dimension rather than excluding it from the narrative.

To address concerns regarding network directionality and technical rigor, we introduced directed centrality measures, including PageRank and HITS, and expanded our cohesion tests to incorporate bicomponent analysis and average path lengths. These additions confirm that our original findings regarding the structural prominence of powerful clans remain consistent

across both directed and undirected representations. Furthermore, we have significantly improved the manuscript's readability by redesigning Figure 2 for greater analytical clarity and relocating the technical mathematical descriptions of the sliding-window procedure to the Supporting Information.

Transparency and reproducibility were also prioritized in this revision. We have elaborated on the potential biases inherent in judicial sources, detailing our stratified sampling strategy and clarifying our protocols for data anonymization and preprocessing. Finally, we standardized the terminology throughout the text and addressed all minor typographical inconsistencies.

We believe these revisions have substantively strengthened the manuscript’s technical rigor and narrative flow. A detailed, point-by-point rebuttal of all reviewer comments is provided in the "Response to Reviewers" document.

Thank you for the opportunity to submit this revised version of our work.

Sincerely,

Reviewer #1:

This is an exceptionally strong paper. The study analyzes the relation between the marriage networks of 'Ndrangheta families and external status measures of the families. It demonstrates convincingly that high-status families occupy more central positions in marriage networks, while their marriages are less important for the networks' overall cohesion. The argument is clear, rigorous, and exciting.

In the introduction, you provide an excellent overview of the specific Mafia group under study and why it is a particularly interesting case to study. You embed the work carefully within prior research and clearly describe the contribution of the analysis. What I found particularly convincing is the way you combined different data sources (including a validation of the external power measure). The data set and the construction of the marriage networks are described with exceptional clarity. The rationales for excluding intra-family marriages, focusing on the largest network component, and restricting the analysis to an unweighted network are transparent and convincing. The analytical strategy is rigorous, supported by a wide range of robustness checks and clear justifications for each analytical decision. This thoroughness gave me great confidence in the robustness of the findings.

The results are fascinating. The way external measures of power relate to families' structural positions in the marriage network is both substantively important and original. The paper is also extremely well-written, making it a pleasure to read. Overall, this is an outstanding contribution, and I recommend acceptance in its current form.

We would like to express our sincere gratitude to the Reviewer for their exceptionally positive and encouraging assessment of our work. We are delighted that the Reviewer found the study's argument rigorous, the methodology transparent, and the findings substantively important. We especially appreciate the recognition of our efforts to combine diverse data sources and maintain clarity in the construction of the marriage networks.

The Reviewer's thoughtful minor comments provided an excellent opportunity to further polish the manuscript. We have carefully addressed each point—specifically by aligning the visualization in Figure 2 with our core centrality measures, expanding the discussion on the strategic versus demographic drivers of bride-groom exchange, and repositioning Figure 4 to better serve as a robustness check. We believe these refinements have strengthened the coherence and narrative flow of the paper. Thank you for the precious indications and for the high appreciation of our study.

I have only a few minor comments that you may want to consider. I would like to emphasize that these are very minor, and the paper is already excellent and convincing as it stands.

Minor comments

- Figure 2: I was surprised to see that this figure uses a different centrality measure than the four introduced earlier. You might consider using one of the previously introduced measures here for consistency.

We thank the reviewer for pointing this out. We agree that Figure 2 should remain fully consistent with the centrality measures introduced and used throughout the analysis. In response (and consistent with the parallel concern raised by Reviewer 3), we revised Figure 2 to align node sizing with a centrality measure already introduced in the paper (eigenvector centrality), and we improved the visualization to enhance interpretability and coherence with the reported statistics. We also increased label sizes and redesigned the figure to be more informative and easier to read. Finally, we will ensure that the final submitted version allows readers to access a high-definition rendering (clickable/zoomable figure) for closer inspection.

- Bride–groom exchange directionality: I was curious to what extent this ratio may be correlated with the (random) gender distribution of children within families, and how much strategic leeway families realistically have. In the discussion, you suggest that some families (like the Papalia family in your example) favor endogamous unions over sending brides to other families. It might strengthen the argument to discuss a bit more explicitly whether this measure is mainly driven by gender ratios or also reflects strategic decisions.

We thank the reviewer for this insightful comment. We agree that bride–groom exchange directionality may be partly shaped by the gender composition of offspring within families, which constrains the range of feasible marital strategies. We have now explicitly acknowledged this caveat in the Discussion section and clarified that intra-family marriages are likely to result from an interplay between demographic constraints and clan-level strategies, rather than from either factor alone. To substantiate the claim that a strategic dimension is also at work, we have expanded the discussion by reporting descriptive evidence on the prevalence of intra-family marriages in the dataset. Specifically, intra-family marriages account for 5.56% of all recorded ties and are considerably more frequent among powerful clans (11.17%) than among other families (4.28%). We also maintain the illustrative comparison between the Barbaro (1/4 of their marriages are between members of the family) and Papalia (we did not record any intra-family marriages), which highlights heterogeneity in intra-family marriage patterns even among powerful clans. On this last point, we note that a typographical error in the earlier version had mistakenly swapped the names of these two families. We apologize; this has now been corrected.

- Figure 4: I found the inclusion of the bride-receiving share on the x-axis somewhat surprising, as this part of the results section is not yet addressing that dimension. Since the only textual reference to this figure (lines 548–550) could also be covered by Table 1, you might consider dropping this figure here, or alternatively explaining its specific purpose and what additional insights it provides.

Thank you for this comment. We agree that introducing the "Bride-Receiving Share" as an x-axis in the "Status" section created an unnecessary load. To address this, we have moved Figure 4 to the Robustness tests section (now Section 2.2.1). This relocation serves two specific purposes and provides insights that Table 1 alone cannot convey: Robustness Check: It visually demonstrates that the structural advantage of powerful clans is not solely a byproduct of one specific marital strategy but is a robust "overhead" that exists across the entire spectrum. The figure reveals also that while the "sweet spot" for centrality is a general structural logic for all clans, the "prestige payoff" is significantly more pronounced for elite families.

In sum, this is a fantastic paper, methodologically rigorous, substantively exciting, and exceptionally well-written. It was fun to review this paper. Thanks!

Thanks, we are glad you appreciated our work and we are encouraged by your positive assessment of the paper's rigor and clarity. We appreciate the time you took to engage so thoroughly with our data and methodology; your suggestions have helped us refine the narrative flow and sharpen our core arguments.

Reviewer #2:

This manuscript presents an analysis of the structural impacts of marital ties among 'Ndrangheta mafia clans. The setting and motivation for the study are interesting and well-described by the authors. The analyses are creative and rigorously detailed; I especially appreciated the authors' careful attention to finding the right counterfactuals and "null" hypotheses for assessing the significance of observed network patterns. As a whole, the study stands to make a solid contribution to the empirical literature on criminal networks.

We are very grateful to the Reviewer for their positive evaluation and for highlighting the rigor of our analytical approach and counterfactual framing. We particularly value the constructive suggestions regarding the edge-removal tests in Figure 5. By expanding our metrics to include the size of the largest bicomponent and average path length, and by incorporating an analysis of edge betweenness, we have been able to provide a much more nuanced and exhaustive picture of network cohesion. We believe these additions have significantly sharpened the empirical contribution of the study. Thank you for these insightful recommendations and for your encouraging words.

Accordingly, I have only very modest recommendations for improving the manuscript, and these recommendations all center on the specific edge-removal tests presented in Figure 5. Here, I actually found the justification for the specific tests carried out to be a little thin. There is not much discussion given to the choice of the two network-structural metrics used to capture network cohesion – largest component (connectivity) and reachability density. To me, these aren't totally satisfying metrics for giving a strong picture of network cohesion, as most networks contain a large connected component and breaking apart this component is often difficult to do. Have the authors considered also testing for the size of the largest connected bicomponent? And for reachability, what about something like the average path length? And while this would be separate from the edge-removal tests, I also think it would be useful to know whether the edges involving powerful clans score higher than the other edges with regard to "edge betweenness."

We agree that the paper can be strengthened further by refining and expanding the edge-removal tests presented in Figure 5, and we are grateful for the specific guidance offered.

In response, we have expanded the rationale and empirical scope of the edge-removal exercise to provide a more comprehensive characterization of network cohesion under targeted edge deletion. In addition to the two original cohesion metrics (largest connected component size and reachability density), we now also report (i) the size of the largest connected bicomponent (as a stricter indicator of cohesion and robustness to fragmentation) and (ii) the average path length (computed within the largest component to ensure interpretability under fragmentation). We also added a complementary analysis, separate from the removal simulations, examining whether alliances involving powerful clans exhibit systematically higher edge betweenness than other alliances.

Congratulations to the authors on a very interesting paper, and hopefully these modest suggestions are helpful in further strengthening it.

Thank you for this thoughtful and encouraging review. We appreciate your positive assessment of the paper's contribution and of our effort to use appropriate counterfactuals and null models.

We believe the implemented additions provide a clearer and more complete picture of network cohesion and the structural role of powerful-clan alliances, strengthening the manuscript in line with your suggestions.

Reviewer #3:

The manuscript examines matrimonial alliances among ’Ndrangheta families using judicial sources and a clan-level marriage network. The authors analyze centrality patterns, gendered exchange dynamics, and network robustness through permutation tests, sliding-window contrasts, and edge-removal simulations. They argue that powerful clans occupy central positions, peripheral-to-peripheral marriages sustain cohesion, and a moderate bride-receiving balance maximizes centrality. The dataset is valuable and the topic important, but several modeling choices, figures, and methodological decisions require clarification.

We are very grateful to the Reviewer for their thorough and constructive critique. Your comments regarding the meso-level abstraction of clans, the importance of directionality, and the nuances of network cohesion metrics have been instrumental in refining our manuscript.

We have addressed the concern regarding 'monolithic units' by providing a stronger sociological justification for clan-level modeling in the 'Ndrangheta context, while also incorporating intra-family endogamy as a distinct strategic dimension. Per your suggestions, we have significantly bolstered our robustness checks by including directed centrality measures (PageRank, HITS) and more granular cohesion tests (bicomponents, average path length). We also appreciate the guidance on streamlining the text and improving our visualizations; the revised multi-panel Figure 2 and the relocated sliding-window technicalities have greatly improved the manuscript’s readability. We believe these revisions have made the study more robust and the findings more transparent.

1) The decision to model clans (surnames) as nodes instead of individuals simplifies the system but removes significant internal heterogeneity, individual-level brokerage, and micro-dynamics. In a context like the 'Ndrangheta, where individuals can play asymmetric roles and intra-clan structures matter, treating each family as a monolithic unit is a strong theoretical assumption. The manuscript should justify this abstraction more fully and acknowledge what information is lost. Alternative modelling approaches (e.g., individual-level networks or bipartite person–clan representations) could produce different insights and should be discussed.

We thank the reviewer for the opportunity to further clarify our approach. We have now explicitly discussed alternative modelling strategies and clarified the rationale for our modelling decision, which was guided by our goal of capturing meso-level, collective strategies of alliance formation rather than individual behavior or intra-family dynamics.

As you suggested, alternative modelling strategies for analyzing matrimonial networks, such as individual-level networks or bipartite person–clan representations, allow the retention of information on intra-clan heterogeneity of roles, individual-level brokerage, and other micro-dynamics. We acknowledge that such models have proven valuable in illuminating these aspects and have generated important insights into the structure of criminal networks and network disruption strategies.

Nevertheless, in the specific empirical context examined here - namely, inter-family marriages in the ’Ndrangheta - we consider modelling clans as nodes to be a more suitable choice. As discussed in the literature reviewed in Section 1.1, the ’Ndrangheta clans are patriarchal blood families, as recruitment occurs primarily through kinship ties and the matrimonial decisions are concentrated at the family level. Consequently, marriages are typically the outcome of strategic decisions taken by families, to which individuals are expected to comply. As a result, matrimonial ties tend to reflect collective family strategies rather than individual decision-making. The clan therefore constitutes – within this specific empirical context – the most relevant unit of analysis and the primary focus of our article.

2) The authors eliminate all marriages between members of the same clan because these do not create inter-clan edges. However, intra-family marriages can be substantively meaningful in mafia genealogies, signalling endogamy, closure, or a strategic preference against external alliances. Excluding them entirely may erase a relevant dimension of matrimonial strategy. Even if self-loops are not used in structural metrics, their frequency and distribution should be reported and incorporated into the substantive interpretation.

We thank the reviewer for this important observation. We agree that intra-family marriages are substantively meaningful in mafia genealogies, as they can signal endogamy, social closure, and strategic preferences for internal consolidation rather than external alliance-making. In response, we refined the rationale for our modelling choices and we now report the frequency and distribution of consanguineous marriages directly in the Methods section. We also incorporate these patterns into the interpretation of the findings, highlighting that powerful clans display a higher propensity for endogamic ties, consistent with strategies of internal cohesion and asset consolidation.

Moreover, rather than being discarded, intra-family marriages could be employed as meaningful weights—either as node-level attributes (indicating family closure) or as tie-strength modifiers—to capture the degree of internal cohesion and reluctance to form external alliances. This alternative would preserve important sociological information without compromising network structure.

At the same time, we maintain our original approach of excluding self-loops from the alliance network used for structural analysis. Our reason is primarily methodological: self-loops do not generate inter-clan connections and therefore cannot contribute to brokerage, bridging, or cohesion between families—i.e., the core relational mechanisms tested in the alliance-based network measures. Retaining them would mechanically inflate degree-based quantities without adding information about cross-family structure. For these reasons, we treat endogamy as an analytically distinct but complementary dimension of matrimonial strategy: we document and interpret it separately, while restricting the network topology to inter-clan ties to preserve coherence with the manuscript’s main research question and network-structural tests.

3) The dataset naturally yields a directed network: the bride's family "sends" a woman and the groom's "receives." Directionality is analytically important for gendered exchange and hierarchy. Despite this, all centrality measures are computed on an undirected version of the network, and directionality is used solely to compute the bride-receiving share. This inconsistency should be addressed. At minimum, the authors should report whether centrality results hold using directed measures (in-/out-degree, directed betweenness, HITS, PageRank). As it stands, it is unclear whether key findings depend on ignoring direction.

Thank you for this important observation. We agree that directionality is substantively meaningful in our setting, and we now address this potential inconsistency explicitly. While our main analyses of cohesion and structural prominence rely on an undirected alliance projection (appropriate for questions about whether a marital tie exists between clans, regardless of who "sends" or "receives" the bride), we have added a directed robustness check computed on the directed marriage network (bride clan to groom clan) restricted to the same largest weakly connected component. Using 5,000 label-permutation tests, we compare powerful versus other clans on in-/out-degree, directed betweenness, PageRank, and HITS scores. The directed results confirm the substantive conclusion that powerful clans are structurally more prominent, and they do not alter our key findings. We report these results in the revised manuscript and discuss why undirected versus directed representations correspond to distinct theoretical questions (alliance existence vs. directional exchange).

In addition, we replicated the "sweet-spot" analysis using directed prestige measures (PageRank and HITS authority) as outcomes, while retaining the same adaptive sliding-window and permutation design. The results corroborate the original pattern: clans with moderately bride-receiving strategies (around 0.55) display the strongest positive contrasts in directed prestige, indicating that the inverted-U relationship is not an artefact of collapsing directionality in the centrality measures.

3) Figure 1, showing the entire clan-level network, is visually dense and difficult to interpret. The collapsing of multi-marriages and the clan-level aggregation produce a nearly unreadable graph with limited analytical value. This figure could be moved to supplementary materials or replaced with more informative visualisations (e.g., cluster-focused subgraphs, regional subnetworks, or edge-type distributions).

We thank the reviewer for this constructive comment. We agree that the original Figure 2 was visually dense and difficult to interpret at the clan-aggregated level. Following this suggestion (and consistent with related remarks raised by Reviewer 1), we revised Figure 2 to improve clarity and increase its analytical value. Specifically, we redesigned the figure into a multi-panel visualization that (i) improves readability by reducing label clutter and increasing font sizes, (ii) highlights the network backbone through a k-core representation, and (iii) adds a tie-composition panel summarizing alliance types by clan status. We believe this revised format is substantially more informative and directly supports the manuscript's key results. In the final

version, the figure will also be provided in high resolution, allowing readers to click and zoom on the image for detailed inspection.

5) Figure 4, based on adaptive sliding-window contrasts, is also difficult to read. The variable window widths, the logic behind the reference bands, and the meaning of the plotted contrasts are not sufficiently explained. Clearer captions and a more explicit walkthrough of how the figure should be interpreted would be helpful.

We have revised the "Analytical Strategy" and the "Robustness" sections to provide an explicit walkthrough of the methodology. Specifically, we now clarify: (1) the logic of the adaptive window (expanding to ensure for stability); (2) the role of the neutral parity point as a reference band; and (3) a more detailed figure caption that guides the reader on how to interpret the curves.

6) The two robustness procedures (equal-count and equal-share removal of edges) are potentially insightful, but the rationale and implementation are not entirely transparent. The manuscript should clarify exactly how edges are classified, how many are removed in each scenario, which structural metrics are monitored, and the logic behind comparing these categories. Additionally, because “elite” and “peripheral” clans are defined using external labels, the dependence of the simulation on these categories should be discussed. The current description makes replication challenging.

Thank you for this suggestion. We agree that the rationale and implementation needed to be clearer for replication, and we revised the Methods accordingly.

First, we now state explicitly how edges are classified. In the undirected clan alliance graph $G = (V, E)$, an edge belongs to E^{pow} if at least one endpoint is a clan labeled as powerful (based on external historical/criminological sources), and to E^{non} otherwise.

Second, we clarify the two removal regimes. In the equal-count procedure, at step k we remove k edges from E^{pow} and k edges from E^{non}, continuing until the smaller subset is exhausted. In the equal-share procedure, we remove the same fraction f of edges within each subset (i.e., $\lfloor f \mid E^{\text{pow}} \mid \rfloor$ and $\lfloor f \mid E^{\text{non}} \mid \rfloor$). In both cases, edges are sampled uniformly at random without replacement, vertices are never removed, and isolated clans remain as singleton nodes.

Third, we specify the cohesion outcomes recorded after each step. In addition to the original largest component size and reachability density, we also monitor the size of the largest biconnected component and the average shortest path length within the largest component.

Finally, we emphasize that powerful clans are defined exogenously and used only to stratify edges. The simulations test whether ties touching powerful clans contribute disproportionately to cohesion relative to other alliances. We also report baseline edge betweenness comparisons across E^{pow} and E^{non} as a complementary structural check.

7) Judicial data are inherently selective and shaped by investigative priorities. While the manuscript acknowledges this, the discussion remains brief. The authors should elaborate on

possible biases (geographical coverage, visibility of certain clans, time periods with heavier prosecution) and how these may influence the structure and interpretation of the marriage network.

We thank the reviewer for this important observation. Accordingly, in Section 4.1 we further articulated the discussion of the limitations associated with the use of judicial sources. In addition, we have expanded the methodological section with further details (also to address the reviewer's suggestion of providing more information on preprocessing of judicial material) that also inform this discussion. Specifically:

We spell out that biased geographical coverage may result in the underrepresentation of families operating in some areas, causing them to appear peripheral or absent in the observed network despite potentially playing a more central role in the underlying relational structure; and differences in visibility among clans may inflate the observed centrality of more closely scrutinized actors and may contribute to a structure that partly reflects investigative attention.

We present the safeguards adopted to mitigate these biases. Among them, we underscore that our sample includes investigations selected through a stratified sampling strategy across the two Calabrian Antimafia Districts Directorates (i.e., Reggio Calabria and Catanzaro). This approach ensures coverage of the entire Calabrian region -- 'Ndrangheta's stronghold -- by proportionally representing investigations conducted under both Directorates, thereby reducing the likelihood that central families were omitted for jurisdictional reasons.

To limit biases arising from differences in clan visibility, we selected 40 investigations across a ten-year time span, a strategy that helps to reduce the influence of case-specific and time-specific investigative priorities.

Regarding the third dimension highlighted by the reviewer—namely time periods characterized by more intense prosecutorial activity—we agree that such periods may affect the visibility of certain ties in judicial data. However, this concern is somewhat less pronounced in the case of matrimonial ties. Judicial documents typically reconstruct extended family relationships across multiple generations, as genealogical information is instrumental for prosecutors in establishing associative ties. As a result, information on marriages often extends beyond the specific generation or time period under investigation, including preceding and subsequent generations. From a network perspective, this reduces the extent to which the observation of matrimonial ties is tightly coupled to specific moments of intensified prosecutorial activity. For this reason, while we acknowledge this potential source of bias, we consider it less critical for the interpretation of matrimonial networks than for other types of relational data. Therefore, to preserve readability, we decided not to discuss it explicitly in the article.

8) The manuscript is notably long and contains methodological detail that could be moved to Supplementary Materials (particularly the mathematical description of the sliding-window method and some robustness checks). Streamlining would significantly improve readability without sacrificing rigor.

We thank Reviewer 3 for this helpful suggestion. We agree that the manuscript can be streamlined by moving highly technical material into Supplementary Materials without reducing transparency or rigor. Accordingly, we revised the paper by relocating (i) the full mathematical description of the sliding-window permutation procedure, (ii) detailed robustness checks and alternative specifications, and (iii) additional technical diagnostics to an ad hoc Annex/Supplement. In the main text, we now provide a shorter, intuition-driven description of the analytical logic and the key inferential steps. This restructuring substantially improves readability while keeping all methodological details fully available for replication.

9) Several references appear tangential, outdated, or not directly relevant. A more selective use of literature—especially regarding classical anthropological sources or reports used as scientific citations—would strengthen the theoretical framing and avoid distraction. I suggest to check these works: https://doi.org/10.3390/math10162929 and https://doi.org/10.1109/TIFS.2023.3256706.

Thank you for your kind suggestions. Accordingly, we:

Rephrased the reference to the DIA report, and the references to the INTERPOL and EUROPOL reports. In the last case we deleted altogether the direct quotations and replaced them with the following wording: "… today is often described by LEAs as one of the most dangerous organized crime groups worldwide";

added references you suggested (https://doi.org/10.3390/math10162929 e https://doi.org/10.1109/TIFS.2023.3256706), along with other relevant works, to further strengthen the theoretical framing of the paper;

shortened the discussion on the anthropological dimension of marriage. However, after careful consideration, we decided to retain at least a brief introductory discussion on the strategic use of marriage across societies and cultures in order to provide sociological context for the reader. We believe this contextualization is important for two reasons. First, the logic of arranged marriages may be relatively distant from contemporary Western societies and therefore benefits from explicit framing. Second, this discussion is consistent with our references to studies on arranged marriages in historical contexts—particularly those involving the Florentine elite—which we draw upon in the discussion of the findings. Overall, we believe that providing the reader with a basic frame of reference improves the clarity and readability of our analysis.

10) Terminology for "elite," "powerful," and "high-status" clans should be standardized.

We thank the reviewer for pointing this out. Following her suggestion, we standardized the terminology throughout the manuscript and now consistently use the term powerful to refer to elite/high-status clans. Exceptions were made when the term elite is used in reference to

studies on the Florentine elite and other literature, in order to remain consistent with the terminology adopted by the authors.

11) The Data Availability Statement is adequate, but a brief note on anonymization or preprocessing of judicial material would enhance transparency.

We thank the reviewer for raising this point and for the opportunity to further enhance the transparency of our study. To this end, we added a brief description of the selection and preprocessing of judicial material to the methodological section of the article.

We specified that the 40 judicial documents were randomly selected from a database we created of all criminal investigations (n. 192) conducted under Article 416-bis against the 'Ndrangheta in Calabria. These investigations were mentioned in DIA reports (Antimafia Investigative Directorate, the Italian multi-force law enforcement agency specialized in anti-mafia investigations) issued between 2007 and 2016. From this set, we randomly selected a sample of 40 investigations, stratified according to the distribution of investigations between the two DDAs. In particular, 25 of the selected investigations were led by the DDA of Reggio Calabria and 15 by the DDA of Catanzaro. We then retrieved the associated judicial documents, which were kindly provided by the Milan-based DIA. Finally, data extraction and coding were carried out manually, using ten keywords corresponding to Italian terms for family members. We also conducted consistency checks on individuals, marriages, and families, consolidating duplicate entries for both individuals and families.

Moreover, we added the following sentence on anonymization to the Data Availability Statement: In accordance with the PLOS ONE data sharing policy, we have made the matrimonial network publicly available to enhance transparency and replicability. The names of 'Ndrangheta families have been anonymized in order to comply with relevant ethical and legal considerations. In the dataset, powerful clans are identified with the letter 'P'.

12) A final proofreading pass could remove minor typographical inconsistencies.

We carefully checked our manuscript for inconsistencies, typos, grammar errors.

6. PLOS authors have the option to publish the peer review history of their article (what does this mean?). If published, this will include your full peer review and any attached files.

If you choose “no”, your identity will remain anonymous but your review may still be made public.

Do you want your identity to be public for this peer review? For information about this choice, including consent withdrawal, please see our Privacy Policy.

Reviewer #1: No

Reviewer #2: No

Reviewer #3: No
